# Preparation of Graphite Phase g-C_3_N_4_ Supported Metal Oxide Activator and Its Performance in Activating Peroxodisulfate Degradation of Methyl Orange

**DOI:** 10.3390/ijerph192416651

**Published:** 2022-12-11

**Authors:** Nan Yang, Zhihan Zhang, Shicheng Zhang, Liting Chen, Jia Zhu, Jingsi Gao

**Affiliations:** 1 School of Materials and Environmental Engineering, Shenzhen Polytechnic, Shenzhen 518055, China; 2 College of Chemical Engineering, Beijing University of Chemical Technology, Beijing 100029, China; 3 Shi Jia Zhuang Municipal Design & Research Co., Ltd., Shijiazhuang 050000, China

**Keywords:** Ag_2_O/g-C_3_N_4_, dye wastewater, pseudo-first-order kinetic, activation reaction, free radicals

## Abstract

In order to improve the catalytic activity and recycling performance of semiconductor activators, and improve the activation pathway of persulfate, graphitic carbon nitride (g-C_3_N_4_) was prepared by calcining melamine, and a composite activator Ag_2_O/g-C_3_N_4_ based on g-C_3_N_4_ supported metal oxide was prepared using a precipitation method. The morphology, structure, and basic properties of the composites were characterized using SEM, XRD, FT-IR and XPS. The activation efficiency of the Ag_2_O/g-C_3_N_4_ composite activator on peroxodisulfate (PDS) was explored. The results showed that Ag_2_O in the composite activator was highly dispersed on the surface of g-C_3_N_4_ and did not change the molecular structure of g-C_3_N_4_ significantly. Under different activation systems, the degradation process of MO was best fitted under the pseudo-second-order reaction kinetic model, compared to the separate g-C_3_N_4_ or Ag_2_O activated PDS systems; the activation of the PDS system with Ag_2_O/g-C_3_N_4_ had the best effect on MO degradation; and the composite activator Ag_2_O/g-C_3_N_4_ showed better activation performance. Under the conditions that the mass combined ratio of Ag_2_O in the activator was 12%, the initial concentration of PDS was 4 mmol/L, the initial concentration of the activator was 1.25 g/L, and the initial pH was 3, the degradation degree of MO reached 99.4% after 40 min reaction. The free radical quenching experiment proved that the active substances that could degrade MO in the system were SO_4_^−^· and ·OH, and the effect of SO_4_^−^· was greater than that of ·OH. The degradation degree of MO in the reaction system remained above 80% after four cycles of use, and the crystal structure of Ag_2_O/g-C_3_N_4_ did not change significantly before and after the reaction. The above results show that Ag_2_O/g-C_3_N_4_ is an efficient and stable composite activator with good application potential in the treatment of dye wastewater by activating PDS.

## 1. Introduction

Water is one of the natural resources that people depend on for survival. In recent years, due to the rapid development of industry, many refractory organic pollutants have emerged, among which dye is one of the refractory organic pollutants. Dye wastewater has the characteristics of high chroma, poor biodegradability, and substantial toxicity [1]. Untreated direct emissions could seriously damage the ecological environment and threaten human health [2]. Methyl orange (MO) is a typical azo dye. At present, there are still many deficiencies in the research on the degradation of MO, for example, photocatalysis requires additional running devices, which increases processing costs [3]. The catalytic wet oxidation method requires high reaction conditions [4], while the biological process requires a long reaction time and the treatment effect is unstable [5]. However, the advanced oxidation of persulfate has attracted wide attention due to its advantages of strong oxidation capacity, high removal efficiency, and low cost.

Persulfate (PS) is a kind of oxidant commonly used in the treatment of organic wastewater, including peroxymonosulfate (PMS) and peroxydisulfate (PDS) [6]. The sulfate radical (SO_4_^−^·) produced by PS can oxidize and degrade most organic pollutants [7]. Compared to hydroxyl radical (·OH) generated by peroxide oxidation, SO_4_^-^ has a higher redox potential and a wider pH application range [8,9], and is widely used in the treatment of refractory wastewater. However, the ability of PS itself to produce free radicals is weak; in order to enhance its oxidative degradation ability of organic pollutants, it is necessary to activate a way to make it produce more free radicals. Hua et al. [10] used applied DC voltage-activated PDS to treat tetrabromobisphenol A (TBBP-A) production wastewater, and the results showed that acidic conditions were conducive to the degradation of TBBP-A by electrically activating PDS. Under the system’s optimal condition, the TOC degradation degree of wastewater reached more than 40% in about 1 h. Chen et al. [11] studied the heterogeneous activation of granular activated carbon to degrade acid orange Ⅱ (AOⅡ) by different persulfates and found that PDS was more effective than PMS, and the degradation degree of AOⅡ reached 91.7% under the optimal condition of the system. The current ways of activating PS have problems, such as fast system loss, high energy consumption, and secondary pollution caused by toxic substances produced by the prone of unexpected nitration processes [12,13]. Therefore, searching for an efficient and environmentally friendly activation method has certain research significance.

Graphite phase carbon nitride (g-C_3_N_4_) is a kind of non-metallic polymer semiconductor with the advantages of easy synthesis, high thermal and chemical stability, green and non-toxic, etc. In recent years, it is often used as an activator in the field of advanced oxidation treatment of hard-to-degrade organics [14,15,16]. However, g-C_3_N_4_ suffers from defects such as a small specific surface area and relatively large band gap width, which limit its application in the field of catalysis. Modifying g-C_3_N_4_ through morphological modulation, elemental doping, and the construction of heterostructures can effectively increase its specific surface area and improve the performance of g-C_3_N_4_. Compared to other semiconductor materials, silver-based semiconductors have a narrow band gap and can absorb visible light. Silver oxide (Ag_2_O) is a semiconductor material with a cubic structure, which exists in the form of brownish-black powder; it is non-toxic and has good visible light response performance, and is often used in the field of photocatalytic degradation of pollutants in water [17]. However, Ag_2_O has poor stability and can easily aggregated and reduced to elemental Ag under light conditions. In order to improve the stability of Ag_2_O, it can be combined with g-C_3_N_4_ to increase the specific surface area of g-C_3_N_4_ and improve the dispersion of Ag_2_O, so that a similar synergistic effect can be formed between Ag_2_O and g-C_3_N_4_.

In fact, Ag_2_O/g-C_3_N_4_ composites have been widely used in the field of photocatalysis with a good degradation effect on pollutants [18,19], and Ag^+^ activated persulfate has also been recognized by most scholars. In this study, Ag_2_O/g-C_3_N_4_ composites were prepared successfully using a liquid-phase precipitation method with g-C_3_N_4_ as the carrier loaded with Ag_2_O, and the morphological structure and basic properties of the Ag_2_O/g-C_3_N_4_ composites were characterized using SEM, XRD, FT-IR and XPS. Taking methyl orange (MO) as the simulated dye wastewater, the effects of different activation systems, the mass composite ratio of Ag_2_O in Ag_2_O/g-C_3_N_4_, the initial concentration of PDS, the initial concentration of the activator, and the initial pH value on the degradation of MO were investigated, and the efficiency of the activator Ag_2_O/g-C_3_N_4_ in activating PDS to degrade MO was also investigated. At the same time, the activation mechanism was discussed, and the stability of the activator was verified by a cycling experiment and XRD spectra before and after the activator reaction. This study is expected to solve the shortcomings of the existing semiconductor catalysts and the activation mode of peroxydisulfate and provide a reference for the further application of composite semiconductor materials.

## 2. Materials and Methods

### 2.1. Chemicals

Melamine and tert-Butanol (TBA) were purchased from Aladdin Biochemical Technology Co., Ltd. (Shanghai, China). Sodium hydroxide (NaOH) and AgNO_3_ were purchased from Macklin Biochemical Technology Co., Ltd. (Shanghai, China). Methyl orange (MO), methanol (MeOH), sodium persulfate (PDS), and sulfuric acid (H_2_SO_4_) were purchased from Tianjin Damao Chemical Reagent Co., Ltd. (Tianjin, China). The above reagents were analytically pure.

### 2.2. Preparation of Activator Ag_2_O/g-C_3_N_4_

#### 2.2.1. Preparation of g-C_3_N_4_

A total of 10.0 g of melamine was accurately weighed and evenly placed in the corundum ark of the quartz tube of the tube furnace, the mixture gas (79% N_2_ + 21% O_2_) was slowly passed into the ark, and the temperature was increased to 550 °C at a rate of 5 °C/min and held for 4 h. The resulting yellow solid was thoroughly pulverized in an agate mortar to obtain g-C_3_N_4_ solid powders.

#### 2.2.2. Preparation of Ag_2_O

Without adding g-C_3_N_4_, a certain amount of NaOH solids was weighed in 50 mL of deionized water for magnetic stirring. After 30 min, a certain amount of AgNO_3_ solids was added in the dark, and the brown precipitate was obtained by continuous stirring for 30 min. Then, the reaction solution was centrifuged, washed, and dried. The dried black solids were collected to obtain Ag_2_O particles and stored away from light.

#### 2.2.3. Preparation of Ag_2_O/g-C_3_N_4_

A certain amount of g-C_3_N_4_ and NaOH solids was weighed in 50 mL of deionized water, stirred for 30 min at room temperature, and then a certain amount of AgNO_3_ solids was added away from light. The mixture was stirred continuously for 30 min, and the brown precipitate was obtained by centrifugation, separated, and washed with deionized water. The solid product was dried at 60 °C and collected, which was the compound activator. It was recorded as x% Ag_2_O/g-C_3_N_4_ (x represents the mass ratio of Ag_2_O in the solid activator, %), and stored away from light.

### 2.3. Experiment Methods

A MO solution was used as a mock dye effluent with an initial concentration of 20 mg/L and a solution volume of 50 mL. The pH required for the reaction was adjusted with 0.1 mol/L of dilute sulfuric acid solution and 0.1 mol/L of sodium hydroxide solution. Unless otherwise specified, the initial concentration of PDS was 4 mmol/L, the activator concentration was 1.0 g/L, and the reaction time was 60 min.

A certain amount of the activator was weighed and added into the beaker containing the MO solution, and the adsorption-desorption equilibrium was reached by magnetic stirring for 30 min. Then, a certain amount of PDS was weighed and added into the reaction system for the activation degradation experiment of MO. The sample was taken when the reaction was carried out until a specific time and filtered by a 0.45 μm aqueous filter head, and the absorbance of the sample was measured at 465 nm wavelength. In order to exclude the influence of external light on the experimental results, the experiment was conducted in a dark environment, and the ambient temperature was room temperature. The experiments were conducted three times in parallel.

### 2.4. Analysis Methods

The morphology of Ag_2_O/g-C_3_N_4_ was analyzed using a MIRA scanning electron microscope (SEM) from TESCAN, Czech Republic. The working voltage was 1 kV and the magnification was 1000~30,000 times. The crystal structure was analyzed using an X-ray diffractometer (XRD) of the Smart Lab type from Rigaku, Japan, with a scan range from 5° to 80°, a scan speed of 10°/min, a Cu target Kβ line, a tube voltage of 40 kV, and a tube current of 100 mA. The molecular structure was analyzed using an IRAffinity-1s Fourier infrared spectrometer (FT-IR) from Shimadzu, Japan, and the spectral measurement range was 400~4000 cm^−1^. The element valence state was analyzed using an X-ray photoelectron spectrometer (XPS) from Thermo Flyer, Waltham, MA, USA, and the binding energy range was 0~1400 eV. The concentration of MO was determined using a DR3900 ultraviolet spectrophotometer, produced by Hach with a wavelength scanning range of 320~700 nm.

## 3. Results and Discussion

### 3.1. Analysis of Characterization

#### 3.1.1. SEM and XRD Analysis

**Figure 1:**
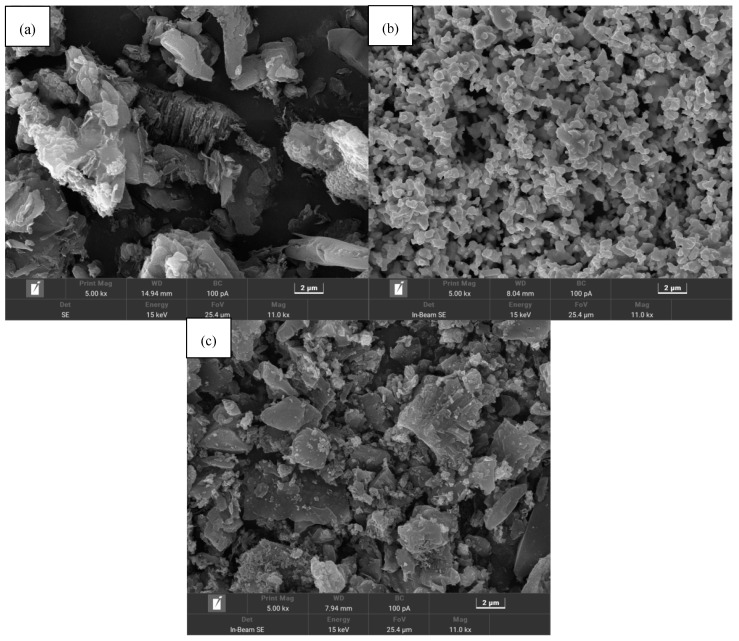
SEM images of g-C_3_N_4_ (**a**), Ag_2_O (**b**), and Ag_2_O/g-C_3_N_4_ (**c**).

The microscopic morphology of the g-C_3_N_4_, Ag_2_O, and Ag_2_O/g-C_3_N_4_ activators were compared using SEM, and the SEM results are shown in Figure 1. As seen in Figure 1a, g-C_3_N_4_ is a blocky structure composed of irregular lamellar aggregates, with a size of 4~8 μm. As seen in Figure 1b, Ag_2_O is formed by the tight aggregation of circular lamellae, with a diameter of 0.5~1 μm. In Figure 1c, Ag_2_O/g-C_3_N_4_ has the lamellar structure of g-C_3_N_4_, and the surface is scattered with fine Ag_2_O particles. These particles do not show the circular lamellar morphology as shown in Figure 1b, due to the low Ag_2_O content in the sample and the high dispersion on the surface of g-C_3_N_4_.

**Figure 2:**
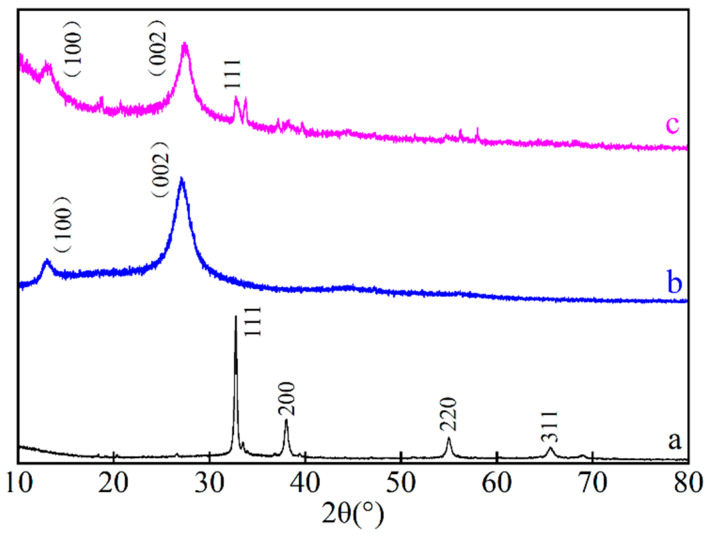
XRD spectrograms of Ag_2_O (**a**), g-C_3_N_4_ (**b**), and Ag_2_O/g-C_3_N_4_ (**c**).

Figure 2 shows the XRD patterns of the Ag_2_O, g-C_3_N_4_, and Ag_2_O/g-C_3_N_4_ activators. It can be seen from Figure 2a that there is a strong characteristic peak at 32.76°, corresponding to the (111) crystal plane of Ag_2_O. The characteristic peaks at 38.04°, 54.99°, and 65.58° correspond to the (200), (220) and (311) crystal planes of Ag_2_O (JCPDS41-1004) [20], respectively. In Figure 2b, the characteristic peaks at 13.38° and 27.55° correspond to the (100) crystal plane and (002) crystal plane of g-C_3_N_4_ (JCPDS87-1526) [21], respectively. As seen in Figure 2c, two characteristic peaks of g-C_3_N_4_ appear at 13.38° and 27.55° for Ag_2_O/g-C_3_N_4_, and at 32.76° for Ag_2_O, indicating that the Ag_2_O/g-C_3_N_4_ complex had been successfully synthesized by the precipitation method. The characteristic peak of Ag_2_O is weaker than that of g-C_3_N_4_, and three weak characteristic peaks of Ag_2_O at 38.04°, 54.99° and 65.58° are not observed. The reason is that the content of Ag_2_O in the complex is low, so these peaks are not detected.

#### 3.1.2. FT-IR Analysis

**Figure 3:**
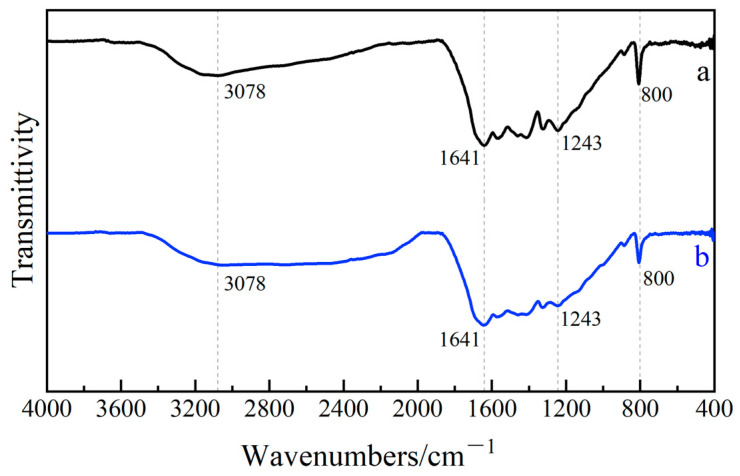
FT-IR spectrograms of g-C_3_N_4_ (**a**) and Ag_2_O/g-C_3_N_4_ (**b**).

Figure 3 shows the FT-IR characterization results of g-C_3_N_4_ and Ag_2_O/g-C_3_N_4_. In Figure 3a, the peak at 800 cm^−1^ is attributed to the stretching vibration of the g-C_3_N_4_ triazine ring unit, the peak at 1243 cm^−1^~1641 cm^−1^ is attributed to the stretching vibration of C-N and C=N structure, and the wide peak at 3078 cm^−1^ is attributed to the stretching vibration of N–H bond, indicating the presence of incomplete melamine in the sample. Figure 3b shows the FT-IR spectrum of Ag_2_O/g-C_3_N_4_. The peak type and peak position of the spectrum are basically consistent with those of Figure 3a, because the content of Ag_2_O in the sample is relatively low, and they also indicate that the loading of Ag_2_O by precipitation does not affect the molecular structure of g-C_3_N_4_.

#### 3.1.3. XPS Analysis

**Figure 4:**
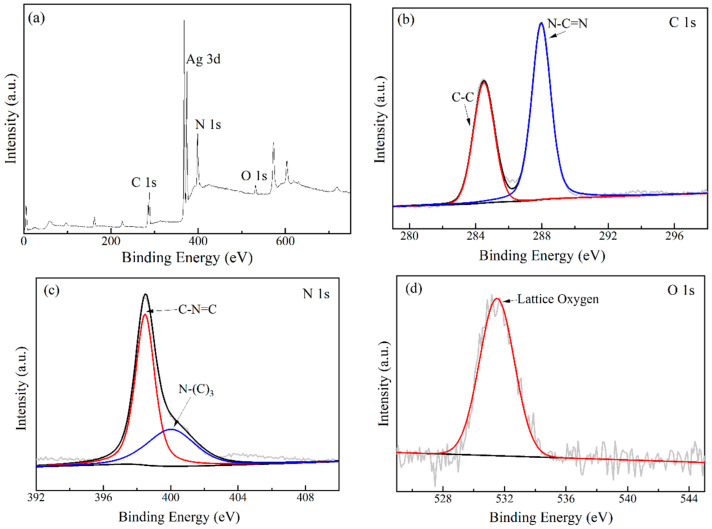

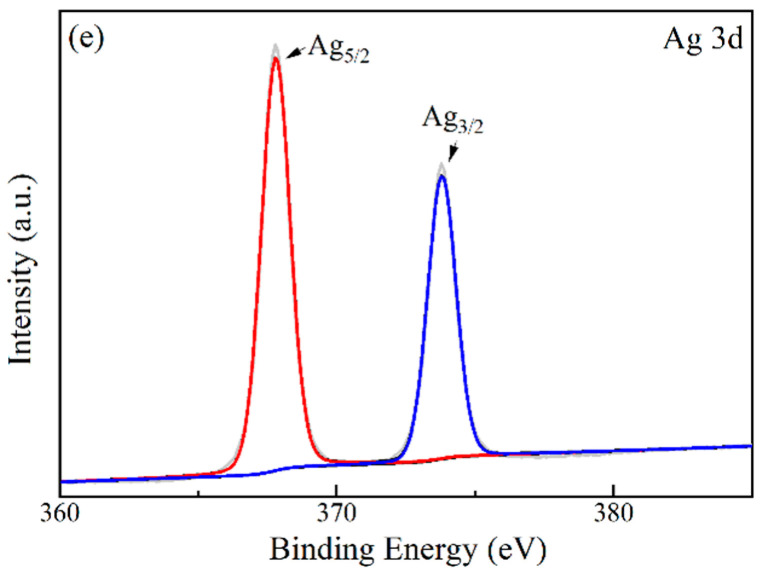
XPS spectrograms of (**a**) all elements, (**b**) C 1s, (**c**) N 1s, (**d**) O 1s, and (**e**) Ag 3d in Ag_2_O/g-C_3_N_4_.

The valence states of each element in the Ag_2_O/g-C_3_N_4_ sample were characterized using XPS, and the results are shown in Figure 4. As seen in Figure 4a, the total spectrum includes the binding energy peaks of four elements, C 1s, N 1s, O 1s, and Ag 3d, indicating that the sample contains four elements, C, N, O, and Ag. In Figure 4b, two binding energy peaks at 284.48 eV and 287.88 eV correspond to C-C in Ag_2_O/g-C_3_N_4_ and sp2 carbon atoms in the structure of nitrogen-containing aromatic ring (n-C = N), respectively. Figure 4c is obtained by fitting and it can be seen that the two binding energy peaks at 398.48 eV and 399.98 eV correspond to the N atom in the triazine structural unit (C = N-C) and the N-(C)3 structure in g-C_3_N_4_, respectively. In Figure 4d, the binding energy peak at 531.48 eV corresponds to lattice oxygen in Ag_2_O. In Figure 4e, the two binding energy peaks at 367.78 eV and 373.78 eV correspond to Ag_5/2_ and Ag_3/2_ [22] in Ag_2_O, respectively. The results of XPS indicate the successful recombination of Ag_2_O and g-C_3_N_4_ in the sample Ag_2_O/g-C_3_N_4_.

### 3.2. Influence of Different Reaction Conditions

#### 3.2.1. Different Activation Systems

**Figure 5:**
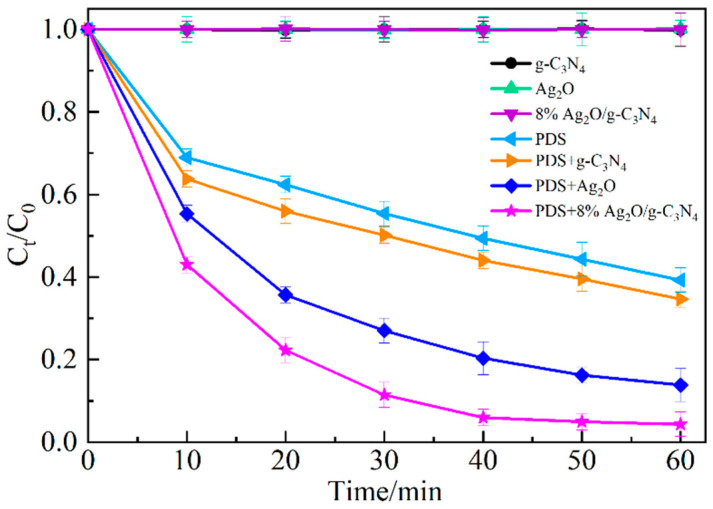
Degradation of MO under different activation conditions.

Figure 5 shows the degradation of MO under different activation systems. Under the conditions of g-C_3_N_4_, Ag_2_O, or Ag_2_O/g-C_3_N_4_ alone, the concentration of MO does not change after the adsorption-desorption equilibrium, indicating that these three materials have no noticeable degradation effect on MO. Under the condition of PDS alone, the degradation degree of MO for 60 min is 60.7%, indicating that the presence of PDS produces some free radicals in the system and has a certain degradation effect on MO. Under the co-existence condition of PDS and g-C_3_N_4_, the degradation degree of MO for 60 min is 65.3%; the degradation degree is only 4.6% higher than that of PDS alone, and there is almost no change in the reaction rate, indicating that g-C_3_N_4_ has little activation effect on PDS. Under the co-existence condition of PDS and Ag_2_O, the degradation degree of MO for 60 min is 86.2%, which is 20.9% higher than that of the g-C_3_N_4_ system co-existing with PDS, indicating that Ag_2_O can activate PDS to produce SO_4_^−^· and improve the degradation degree of MO. However, under the co-existence condition of PDS and 8% Ag_2_O/g-C_3_N_4_, the degradation degree of MO reaches 95.7% after 60 min reaction, which is 9.5% higher than that of the co-existence of the Ag_2_O system with PDS; moreover, it is higher than the degradation degree of MO in the system of the Ag/AgCl/p-g-C_3_N_4_ nanocomposite after 60 min reaction under visible light irradiation [23]. Meanwhile, it greatly saves the reaction time compared with the degradation degree of MO reaches 99.3% after 4 h of reaction [24]. The results indicate that g-C_3_N_4_ loaded with Ag_2_O effectively increases the specific surface area of g-C_3_N_4_ and provides more active sites on the surface of the carrier. At the same time, the dispersion of Ag_2_O on the surface of the carrier is significantly improved and hinders the production of elemental Ag. The activator with 8% Ag_2_O/g-C_3_N_4_ can promote the production of more SO_4_^−^· by PDS and significantly improve the degradation degree of MO.

#### 3.2.2. Reaction Kinetic Analysis

**Figure 6:**
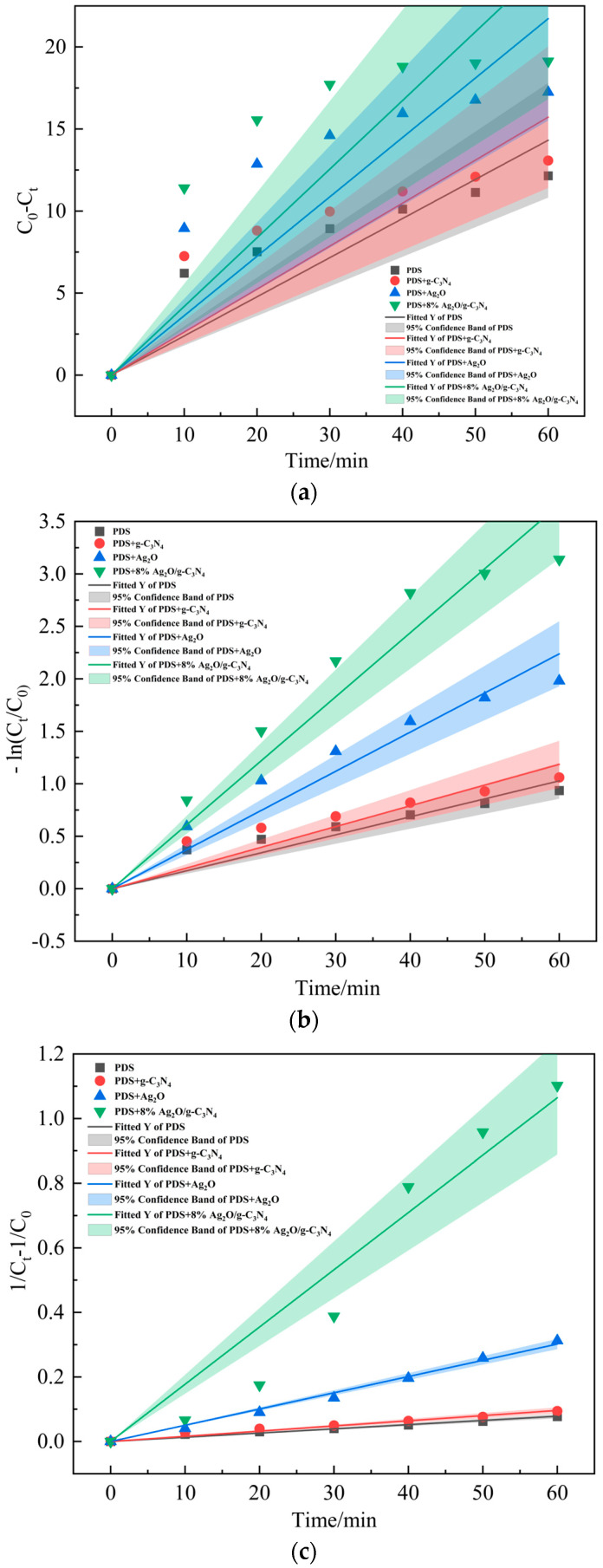

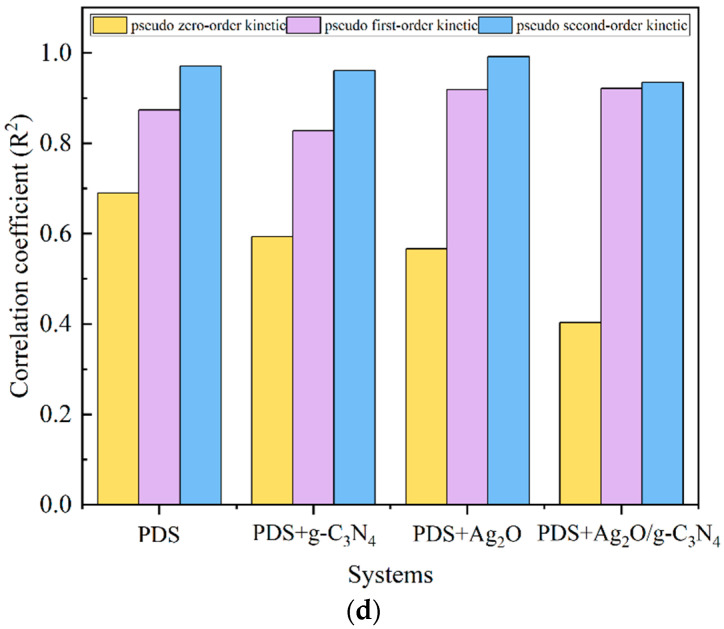
Determination of kinetic rate constants for MO degradation under different activation systems. (**a**) Determination of zero-order kinetic rate constants for MO degradation under different activation systems. (**b**) Determination of pseudo-first-order kinetic rate constants for MO degradation under different activation systems. (**c**) Determination of pseudo-second-order kinetic rate constants for MO degradation under different activation systems. (**d**) Correlation coefficients (R^2^) of different kinetic models of MO degradation under different activation systems.

Figure 6 shows the fitting results of the degradation rate constants of MO under different activation systems. When comparing the fitting results of the different systems under the zero-order kinetic reaction model (Figure 6a), the pseudo-first-order kinetic reaction model (Figure 6b), and the pseudo-second-order kinetic reaction model (Figure 6c), it shows that the fitting results of the degradation process of MO in different systems are better under the pseudo-first-order and pseudo-second-order kinetic reaction models. Figure 6d shows that the correlation coefficients of the different systems under the pseudo-second-order kinetic reaction model are better than those under the pseudo-first-order kinetic reaction model. Table 1 shows the kinetic parameters of the pseudo-first-order and pseudo-second-order reactions of MO under different systems. Table 1b shows that the reaction rate constants of PDS and PDS co-existing systems with g-C_3_N_4_ are 0.0013 L/(mg·min) and 0.0016 L/(mg·min), respectively, indicating that the addition of g-C_3_N_4_ had little effect on the degradation rate of MO. The reaction rate constant of the PDS and Ag_2_O co-existing system is 0.00503 L/(mg·min), which is 2.87 times higher than that of the PDS alone system, indicating that Ag_2_O can activate PDS to promote the degradation of MO. The reaction rate constant increases to 0.01773 L/(mg·min) when PDS and 8% Ag_2_O/g-C_3_N_4_ co-exist, which is 2.52 times higher than that in the co-existence system of PDS and Ag_2_O. It is 0.63 times higher than that of the system of PDS coexisting with 8% Ag_2_O/g-C_3_N_4_ relative to the system of PDS coexisting with Ag_2_O under the pseudo-first-order kinetic reaction model. Thus, 8% Ag_2_O/g-C_3_N_4_ can better activate PDS and accelerate the degradation of MO. The results show that, under the Ag_2_O/g-C_3_N_4_-activated PDS system, the reaction rate constant is the largest and the degradation rate of MO is the fastest, which are consistent with the previous experimental results.

#### 3.2.3. Degradation Reaction Conditions

**Figure 7:**
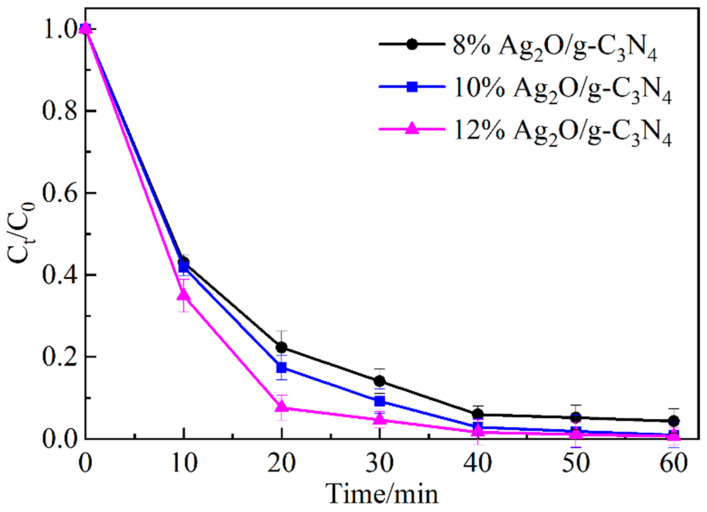
Degradation degree of MO under different Ag_2_O compound ratios.

Figure 7 shows the degradation of MO by Ag_2_O/g-C_3_N_4_ under different Ag_2_O mass composite ratios. The results indicate that the degradation degree increases with an increase in the Ag_2_O mass recombination ratio in the activator. The degradation degree of MO by the activator are 95.7%, 99.1%, and 99.4% within 60 min at 8%, 10%, and 12%Ag_2_O composite ratio, respectively. The degradation degree of MO is the highest when the mass recombination ratio of Ag_2_O is 12%. Thus, 12% Ag_2_O/g-C_3_N_4_ was selected for the subsequent experiment.

**Figure 8:**
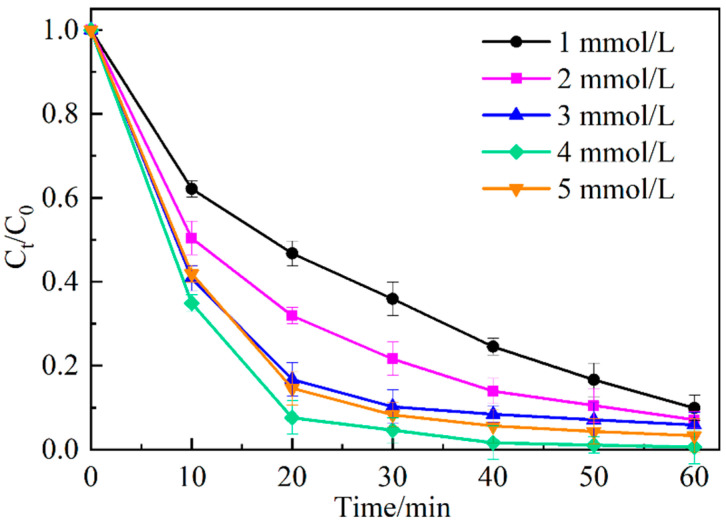
Effect of initial peroxydisulfate concentration on the degradation of MO.

Figure 8 shows the degradation of MO by Ag_2_O/g-C_3_N_4_ at different initial concentrations of PDS. The results indicate that the degradation degree increases from 90.1% to 99.4% when the initial concentration of PDS increases from 1 mmol/L to 4 mmol/L but decreases from 99.4% to 96.7% when the initial concentration of PDS increases from 4 to 5 mmol/L. The reason why the degradation degree increases first and then decreases with the increase of the initial concentration of PDS is that the increase of the concentration of PDS can produce more active substances in the system and promote the faster degradation of MO. However, when the concentration of PDS is too high, the excessive SO_4_^−^· produced in the system leads to the quenching reaction between free radicals. As a result, the concentration of SO_4_^−^· decreases (Equations (1) and (2)) [25]. The initial concentration of PDS was 4 mmol/L for the subsequent experiment. In addition, when the initial concentration of PDS was 4 mmol/L, there was almost no change in the degradation degree of MO after 40 min reaction, so the time of the subsequent experiment was shortened to 40 min.

2SO_4_^−^·→S_2_O_8_^2−^ (1)

SO_4_^−^·+ S_2_O_8_^2−^→SO_4_^2−^ + S_2_O_8_^−^· (2)

**Figure 9:**
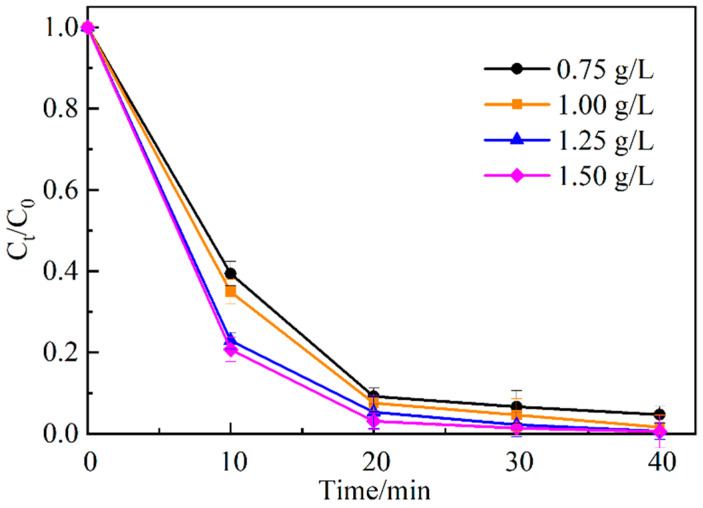
Effect of initial activation concentration on the degradation of MO.

Figure 9 shows the degradation of MO with different initial activator concentrations. As can be seen from the figure, when the initial concentration of the activator increases from 0.75 g/L to 1.25 g/L, the degradation degree of MO at 10 min shows an increasing trend, and the change is more obvious. The degradation degree of MO at 40 min increases from 95.3% to 99.4%. The results show that the increase of the initial concentration of activator increases more active sites for the activation of PDS, and more free radicals are generated in the system, thus improving the degradation efficiency of MO. When the initial concentration of the activator increases from 1.25 g/L to 1.50 g/L, the degradation degree of MO at 40 min only increases by 0.1% to 99.5%. Considering the production cost of the activator, the initial concentration of the activator was 1.25 g/L for the subsequent experiment.

**Figure 10:**
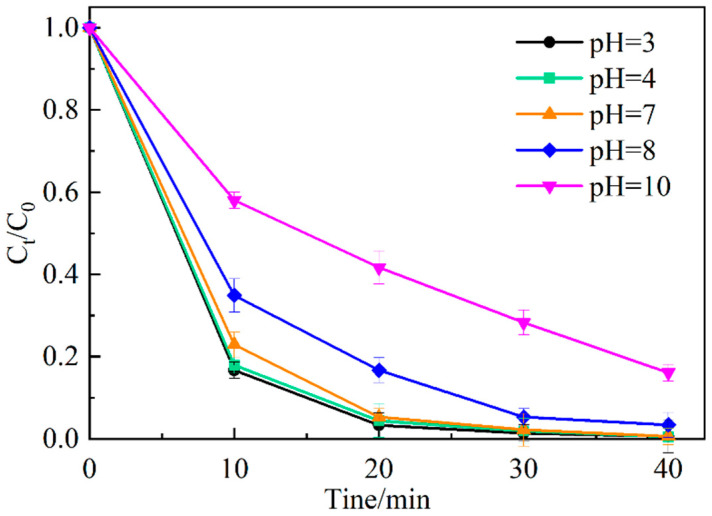
Effect of initial pH value on the degradation of MO.

Figure 10 shows the effect of different initial pH values on the degradation of MO by Ag_2_O/g-C_3_N_4_. As can be seen from the figure, with the initial pH value increasing from 3 to 10, the degradation degree decreases. When the initial pH is less than 7, the degradation degree of MO remains above 99% after 40 min reaction. When the initial pH is greater than 7, the degradation degree of MO is low, and after 40 min, the degradation degree of MO decreases from 96.6% at an initial pH of 8 to 83.9% at an initial pH of 10. The results show that the Ag_2_O/g-C_3_N_4_-activated PDS system has a good removal effect on MO under acidic or neutral conditions. The initial pH value determines the existence form of the active components in the system [26]. Under acidic or neutral conditions, SO_4_^−^· in the system participates in the degradation of MO as the primary active substance, whereas under alkaline conditions, SO_4_^−^· in the system reacts with OH^-^ to generate ·OH with a redox potential lower than that of SO_4_^−^· (Equation (3)). Therefore, the degradation effect of MO under alkaline conditions is worse than that under acidic or neutral conditions [27].

Zhong et al. [28] used g-C_3_N_4_ loaded Fe_3_O_4_ to activate PDS to degrade Rhodamine B. Their experimental results showed that the degradation effect of Rhodamine B was the best under the condition of pH 2.1, and Rhodamine B could be completely degraded within 120 min. The Ag_2_O/g-C_3_N_4_ activator prepared in this study, after optimizing the conditions, activated PDS and could degrade MO with a degradation degree of more than 99% within 30 min or 40 min, significantly shortening the reaction time.

SO_4_^−^·+OH^−^→SO_4_^2−^ +·OH (3)

### 3.3. Detection of Active Species

**Figure 11:**
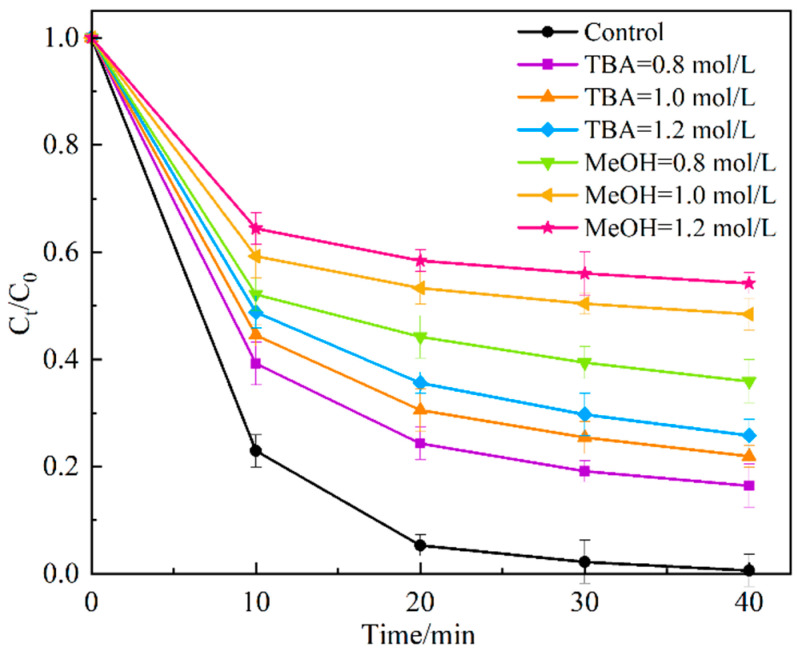
Effect of the quencher on the degradation of MO.

In order to explore the main active substances produced during the activation of PDS by the activator Ag_2_O/g-C_3_N_4_, MeOH and TBA were, respectively, used as the quenchers of SO_4_^−^·and ·OH in the activation system [29]. The concentrations of MeOH and TBA in the control system were 0.8, 1.0, and 1.2 mol/L, respectively. As shown in Figure 11, after TBA has been added, the degradation degree of MO is significantly inhibited, and the inhibition effect becomes more evident with an increase in TBA concentration. After 40 min of reaction, the degradation degree is 83.6%, 78.1%, and 74.2%, respectively, indicating that ·OH is produced in the reaction system and participates in the degradation of MO. After the addition of MeOH, the degradation degree of MO drops sharply, and after 40 min reaction, the degradation degree is 64.1%, 51.6%, and 45.8%, respectively, indicating that SO_4_^−^· is involved in the system of Ag_2_O/g-C_3_N_4_ activated PDS to degrade MO, and the effect of SO_4_^−^· is greater than that of ·OH. The quenching experiments showed that SO_4_^−^· and ·OH are the main active substances in the process of Ag_2_O/g-C_3_N_4_ activation of PDS, and both participate in the degradation of MO, in which SO_4_^−^· plays a more significant role.

### 3.4. Activation Stability of Ag_2_O/g-C_3_N_4_

**Figure 12:**
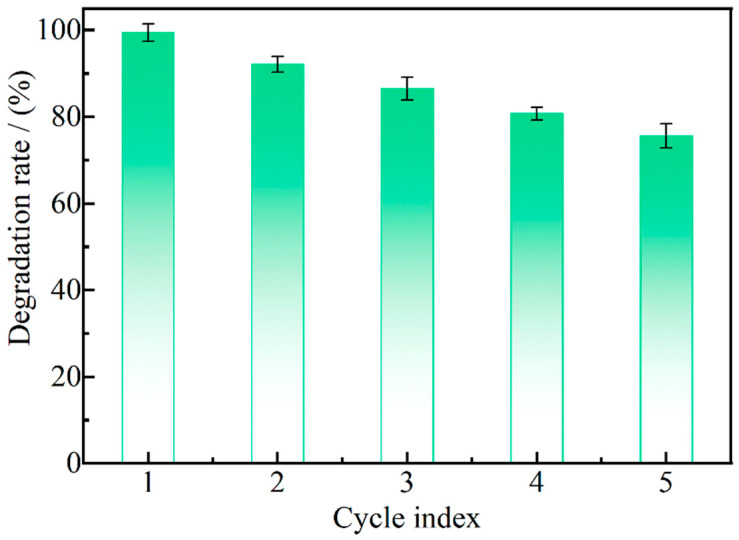
Experiment of stability.

In order to verify the stability of the Ag_2_O/g-C_3_N_4_ activator, five repeated experiments were conducted on the degradation of the Ag_2_O/g-C_3_N_4_ activated PDS, as shown in Figure 12. After four cycles, the degradation degree of MO in the system can still reach 80.7% after 40 min of reaction, and after the fifth cycle experiment, the degradation degree of MO drops to 75.6%. With the increase in the number of experiment cycles, the degradation degree of MO in the reaction system shows a downward trend. The reason may be that, with the progress of the reaction, Ag_2_O in the activator falls off the surface of g-C_3_N_4_, resulting in a decrease in the activation efficiency of the activator to PDS. At the same time, it may also be because, after the end of each cycle experiment, the activator needs to be centrifugated for washing, during which there is an inevitable loss of the activator, which also leads to a decrease in MO degradation degree with an increase in cycle times.

**Figure 13:**
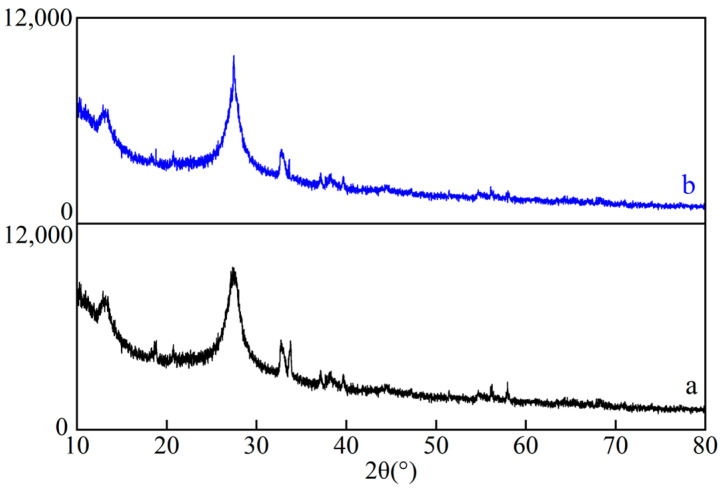
XRD patterns of Ag_2_O/g-C_3_N_4_ before (**a**) and after (**b**) the reaction.

Figure 13 shows the XRD patterns before and after the reaction of the Ag_2_O/g-C_3_N_4_ activated PDS to degrade MO. As can be seen from the figure, there is little difference between the XRD patterns of Ag_2_O/g-C_3_N_4_ before and after the degradation of MO, indicating that the crystal structure of Ag_2_O/g-C_3_N_4_ has not changed significantly. The stability experiment showed that the activator Ag_2_O/g-C_3_N_4_ has considerable stability in the activated PDS system.

## 4. Conclusions

Ag_2_O/g-C_3_N_4_ composited with graphite phase g-C_3_N_4_ as a metal oxide carrier was prepared using a low-cost and safe method. Using SEM, XRD, FT-IR, and XPS, the Ag_2_O/g-C_3_N_4_ composites were successfully synthesized. Ag_2_O in the activator was highly dispersed on the surface of the g-C_3_N_4_ layer in the form of fine particles, and the degradation ability of MO by the composites loaded with Ag_2_O was significantly improved. There was a similar synergistic effect between g-C_3_N_4_ and Ag_2_O. The pseudo-second-order kinetic reaction model was the best fit for the degradation process of MO under different systems. The reaction rate constant of the Ag_2_O/g-C_3_N_4_-activated PDS for MO degradation was significantly higher than that of other systems. Free radical quenching experiments showed that SO_4_^−^· and ·OH were the main active substances in the activation of PDS by Ag_2_O/g-C_3_N_4_, and the effect of SO_4_^−^· was greater than that of ·OH. The degradation degree of MO by the Ag_2_O/g-C_3_N_4_ composites reached 99.4%, which showed stronger catalytic activity and could still maintain more than 80% degradation degree after four cycles of use. The prepared activator Ag_2_O/g-C_3_N_4_ had good catalytic activity and recycling performance.

## Figures and Tables

**Table 1:** Kinetic parameters of reaction for MO degradation under different activation systems. (**a**). Pseudo-first-order kinetic parameters for MO degradation under different activation systems. (**b**). Pseudo-second-order kinetic parameters for MO degradation under different activation systems.

**(a)**		
**Systems**	**Reaction Rate Constant *k*/min^−1^**	**Correlation Coefficient *R*^2^**
PDS	0.01713	0.87383
PDS + g-C_3_N_4_	0.01974	0.82792
PDS + Ag_2_O	0.03730	0.91868
PDS + 8%Ag_2_O/g-C_3_N_4_	0.06097	0.92154
**(b)**		
**Systems**	**Reaction Rate Constant k/L/(mg·min)**	**Correlation Coefficient *R*^2^**
PDS	0.0013	0.97154
PDS + g-C_3_N_4_	0.0016	0.96167
PDS + Ag_2_O	0.00503	0.99184
PDS + 8%Ag_2_O/g-C_3_N_4_	0.01773	0.93537

## Data Availability

The data and materials are available in the manuscript.
